# Functional analysis of Flavonoid 3′,5′-hydroxylase from Tea plant (*Camellia sinensis*): critical role in the accumulation of catechins

**DOI:** 10.1186/s12870-014-0347-7

**Published:** 2014-12-10

**Authors:** Yun-Sheng Wang, Yu-Jiao Xu, Li-Ping Gao, Oliver Yu, Xin-Zhen Wang, Xiu-Juan He, Xiao-Lan Jiang, Ya-Jun Liu, Tao Xia

**Affiliations:** Key Laboratory of Tea Biochemistry and Biotechnology, Ministry of Education in China, Anhui Agricultural University, Hefei, Anhui China; School of Life Science, Anhui Agricultural University, Hefei, Anhui China; Conagen Inc, 15 DeAngelo Dr, Bedford, MA 01730 USA; Wuxi NewWay, 401 Xing Yuan Bei Road, Wuxi, Jiangsu China

**Keywords:** *Camellia sinensis*, Flavonoid 3′5′-hydroxylase, Functional analysis, Heterologous expression, Catechins

## Abstract

**Background:**

Flavonoid 3′,5′-hydroxylase (F3′5′H), an important branch point enzyme in tea plant flavan-3-ol synthesis, belongs to the CYP75A subfamily and catalyzes the conversion of flavones, flavanones, dihydroflavonols and flavonols into 3′,4′,5′-hydroxylated derivatives. However, whether B-ring hydroxylation occurs at the level of flavanones and/or dihydroflavonols, *in vivo* remains unknown.

**Results:**

The *Camellia sinensis F3′5′H* (*CsF3′5′H*) gene was isolated from tea cDNA library. Expression pattern analysis revealed that *CsF3′5′H* expression was tissue specific, very high in the buds and extremely low in the roots. *CsF3′5′H* expression was enhanced by light and sucrose. Over-expression of *CsF3′5′H* produced new-delphinidin derivatives, and increased the cyanidin derivative content of corollas of transgenic tobacco plants, resulting in the deeper transgenic plant flower color. Heterologous expressions of CsF3′5′H in yeast were carried out to demonstrate the function of CsF3′5′H enzyme *in vitro*. Heterologous expression of the modified *CsF3′5′H* (*CsF3′5′H* gene fused with *Vitis vinifera* signal peptide*,* FSI) revealed that 4′-hydroxylated flavanone (naringenin, N) is the optimum substrate for CsF3′5′H, and was efficiently converted into both 3′4′- and 3′4′5′-forms. The ratio of 3′4′5′- to 3′4′-hydroxylated products in *FSI* transgenic cells was significantly higher than *VvF3′5′H* cells.

**Conclusions:**

CsF3′5′H is a key controller of tri-hydroxyl flavan-3-ol synthesis in tea plants, which can effectively convert 4′-hydroxylated flavanone into 3′4′5′- and/or 3′4′-hydroxylated products. These findings provide animportant basis for further studies of flavonoid biosynthesis in tea plants. Such studies would help accelerate flavonoid metabolic engineering in order to increase B-ring tri-hydroxyl product yields.

**Electronic supplementary material:**

The online version of this article (doi:10.1186/s12870-014-0347-7) contains supplementary material, which is available to authorized users.

## Background

Flavonoids are polyphenol antioxidants found naturally in plants, which possess key pharmacological activities, including antioxidant, antimutagenic, anticarcinogenic, and antibacterial properties [1]. Flavonoids in most higher plants can be divided into six major subgroups: chalcones, flavones, flavonols, flavan-3-ols (catechins), anthocyanins, and proanthocyanins (PAs, also called condensed tannins, flavan-3-ol and flavan-3,4-diol polymers) [2].

The structure of the flavonoid B ring is the primary determinant of the antioxidant activity of flavonoids [3], and flavonoids can be divided into three subclasses according to the hydroxylation pattern of their B-ring, including B-ring 4′-hydroxylated, 3′4′-dihydroxylated, and 3′4′5′-trihydroxylated compounds. The number of hydroxyl groups on the B-ring affects the capacity to inhibit lipid peroxidation [4,5]. For instance, Liu and Yang reported that the antioxidant activity of epigallocatechin-3-gallate (EGCG) is greater than that over epigallocatechin (ECG) at concentrations of up to 100 mg · L^−1^ [6].

**Figure 1:**
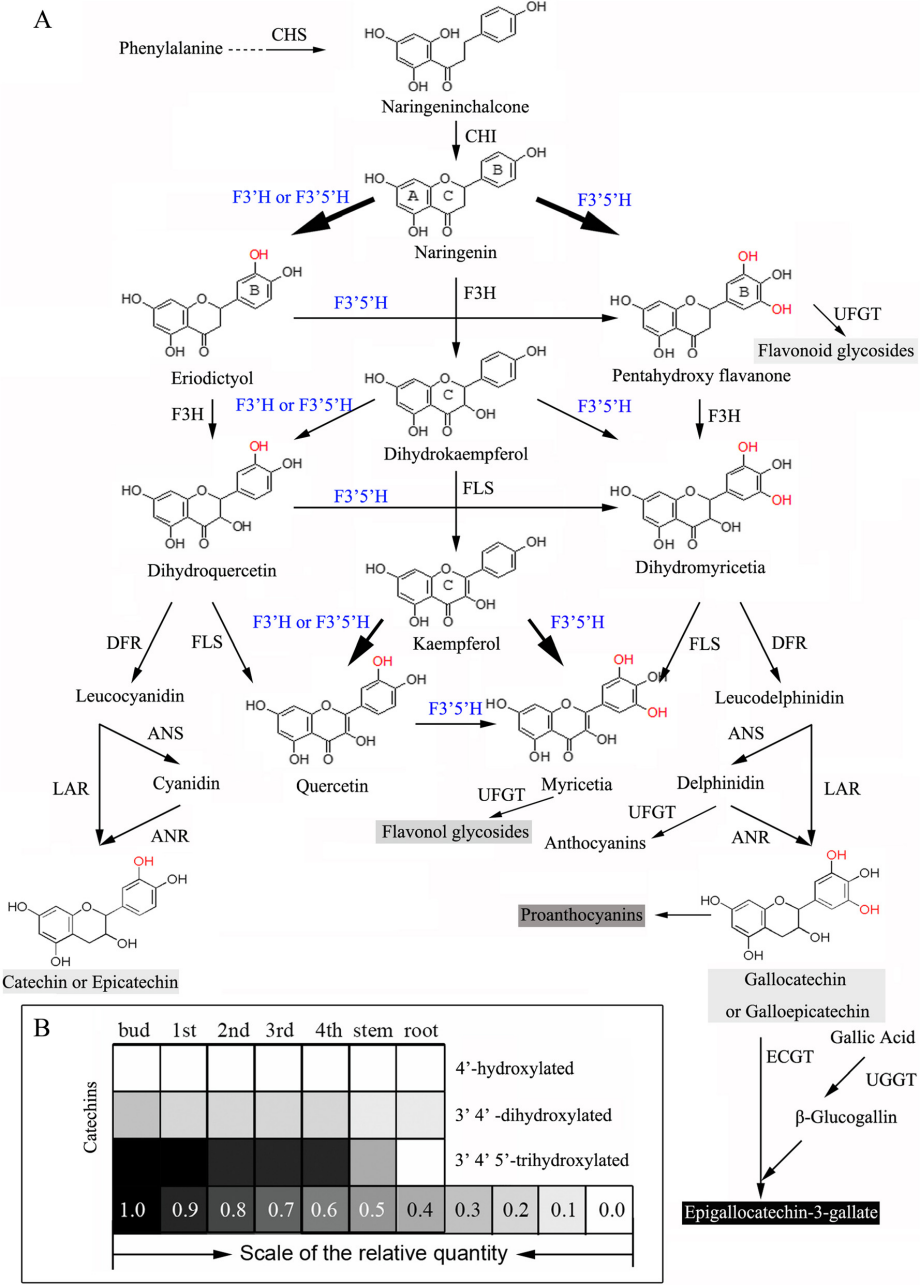
**Biosynthesis pathway and end-product accumulation of flavonoids in** ***camellia sinensis*** **. (A)** Biosynthesis pathway of flavonoids. CHS, chalcone synthase; CHI, chalcone isomerase; F3H, flavanone 3-hydroxylase; F3′H, flavonoid 3′-hydroxylase; F3′5′H, flavonoid 3′,5′-hydroxylase; DFR, dihydroflavonol 4-reductase; FLS, flavonol synthase; LAR, leucoanthocyanidin reductase; ANS, anthocyanidin synthase; ANR, anthocyanidin reductase; UFGT, UDP-glycose flavonoid glycosyltransferase; UGGT, UDP-glucose galloyl-1-O-β-D-glucosyltransferase; ECGT, epicatechins: 1-O-galloyl-β-D-glucose O-galloyltransferase; **(B)** Relative quantity of different flavonoid compounds. The data for relative quantity of different flavonoid compounds were quoted from Jiang (Jiang XL, 2013).

In the flavonoid biosynthesis pathway, the hydroxylation pattern of the B-ring is determined by two cytochrome P450-dependent monooxygenases (P450s): flavonoid 3′-hydroxylase (F3′H) and flavonoid 3′,5′-hydroxylase (F3′5′H). Hydroxylation of the 5′-position by F3′5′H is a particularly important step, which determines the B-ring tri-hydroxyl flavonoid end-product (EGCG or delphinidin) formed in plants, as illustrated in Figure 1.

*F3′5′H*s have been previously cloned and functionally analyzed from multiple plants, including grape (*Vitis vinifera*) [7,8], petunia (*Petunia hybrida*), snapdragon (*Antirrhinum majus*) [9], Cineraria (*Pericallis hybrida*) [10], tomato (*Solanum lycopersicum*) [11], big leaf periwinkle (*Vinca major*) [12], and potato (*Solanum tuberosum*) [13]. Through heterologous expression in transgenic plants and yeasts, F3′5′Hs were shown to hydroxylate a broad range of flavonoid substrates, including naringenin (N), dihydrokaempferol (DHK), kaempferol (K) and apigenin [8,14]. However, optimum substrates for these enzymes remain to be determined.

Tea (*Camellia sinensis*) is an important commercial crop, the leaves of which can be processed into popular nonalcoholic beverages. Because of the high flavonoid content, epidemiological and pathological studies have suggested that tea consumption may potentially be protective against human cancers [15,16] and high blood pressure [17], and contribute to weight reduction [18]. The total concentration of flavonoid compounds is around 12–24% of tea leaf dry mass [19]. We have previously shown that catechins are among the most abundant flavonoids in tea leaves, followed by proanthocyanidins (PAs), flavonols, flavones and anthocyanins (Figure 1A) [20,21]. In recent years, some of the flavonoid structural and regulatory genes have been cloned, and functions of these genes have been investigated [22-25].

While 4′-hydroxylated catechins are very rare or undetectable in tea leaves [22], 3′4′5′-trihydroxylated catechins (gallocatechin (GC), EGC, and EGCG), are the most abundant flavonoids in young leaves and the stem, with significantly higher concentrations than 3′4′-dihydroxylated catechins (catechin (C), epicatechin (EC) and ECG) (Figure 1B). Therefore, characterizing the pattern of B-ring hydroxylation is clearly a valuable contribution to the understanding of flavonoid biosynthesis in tea plants.

However, it has not yet been possible to prepare active membrane-bound F3′5′H enzymes from *Camellia sinensis*, and it is still unclear whether B-ring hydroxylation occurs at the level of flavanones and/or dihydroflavonols, *in vivo*. Aiming to analyze the *in vivo* expression pattern of CsF3′5′H and to characterize the function of this gene *in vitro*, we isolated the *CsF3′5′H* gene from tea cDNA library. We found that *CsF3′5′H* was highly expressed in the bud, but little or no *CsF3′5′H* was detected in the root. *CsF3′5′H* expression was enhanced by light and sucrose treatment, and over-expression of *CsF3′5′H* resulted in production of delphinidin derivatives, producing redder flowers in transgenic tobacco plants, in comparison to with wild type. Heterologous expression of modified *CsF3′5′H* in yeast revealed that 4′-hydroxylated flavanone (naringenin, N) is the optimum substrate for CsF3′5′H, and the ratio of 3′4′5′- to 3′4′-hydroxylated products in the modified *CsF3′5′H* transgenic cells was significantly higher than in *VvF3′5′H* cells.

## Results

### Isolation and characterization of the *CsF3′5′H* gene

**Figure 2:**
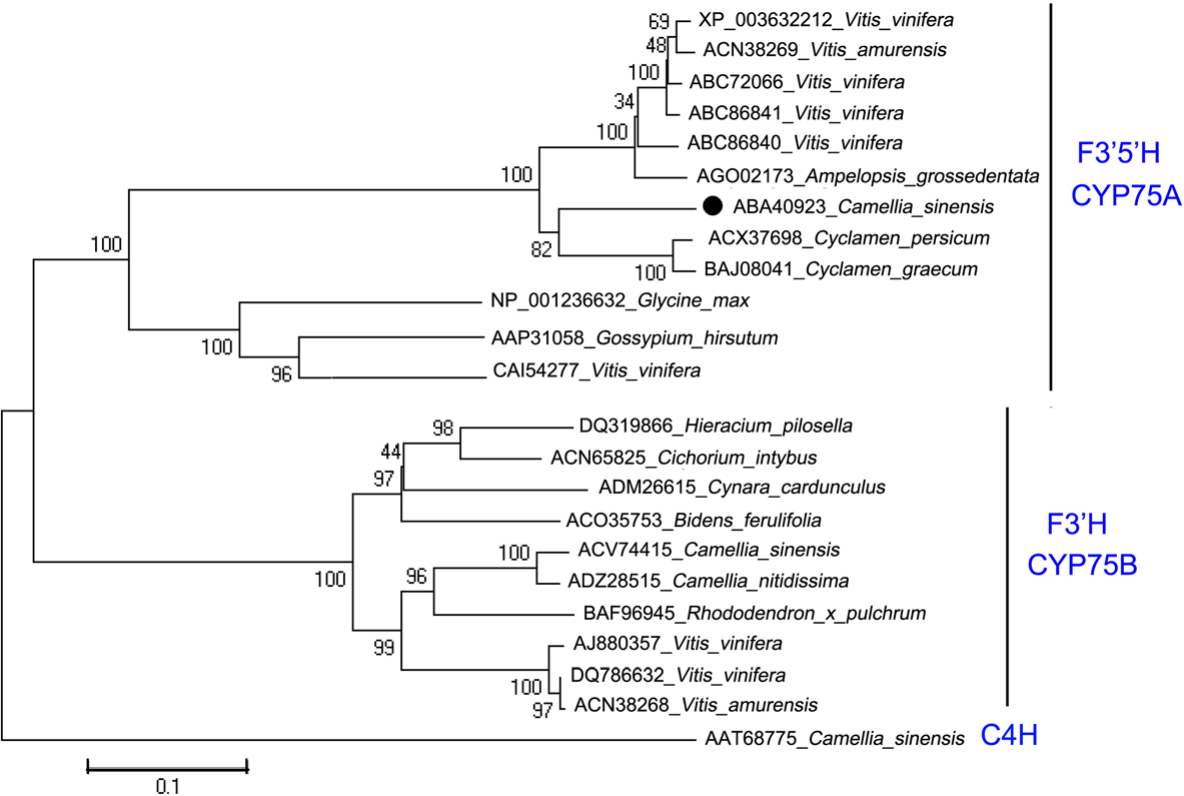
**Phylogenetic tree for a selection of F3′5′H protein.** Phylogenetic tree based on amino acid sequences of F3′Hs and F3′5′Hs in various plant species from the NCBI web page. Accession numbers are displayed in the figure. Bootstrap values (1,000 replicates) are shown at nodes.

The Cs*F3′5′H* gene (NCBI cDNA accession number: DQ194358, protein number: ABA40923) was successfully cloned from the cDNA library of the 3rd tea leaf, and encoded 510 amino acid residues. A BLAST search (NCBI) performed with the coding sequence revealed 83, 82 and 81% identity with *Cyclamen persicum* (ACX37698)*, Cyclamen graecum* (BAJ08041) and *Vitis vinifera* (XP_003632212) genes, respectively. The phylogenetic tree (Figure 2) was generated using protein sequences from several plant F3′5′H and F3′H enzymes retrieved from the NCBI database. The tree demonstrated that F3′Hs and F3′5′Hs were grouped in CYP75B and CYP75A clusters, respectively. CsF3′5′H was grouped into the CYP75A subfamilies, and most closely related to the F3′5′H enzymes of *Cyclamen persicum*, *Cyclamen graecum* and *Vitis vinifera*.

### Expression pattern of *CsF3′5′H* in tea

**Figure 3:**
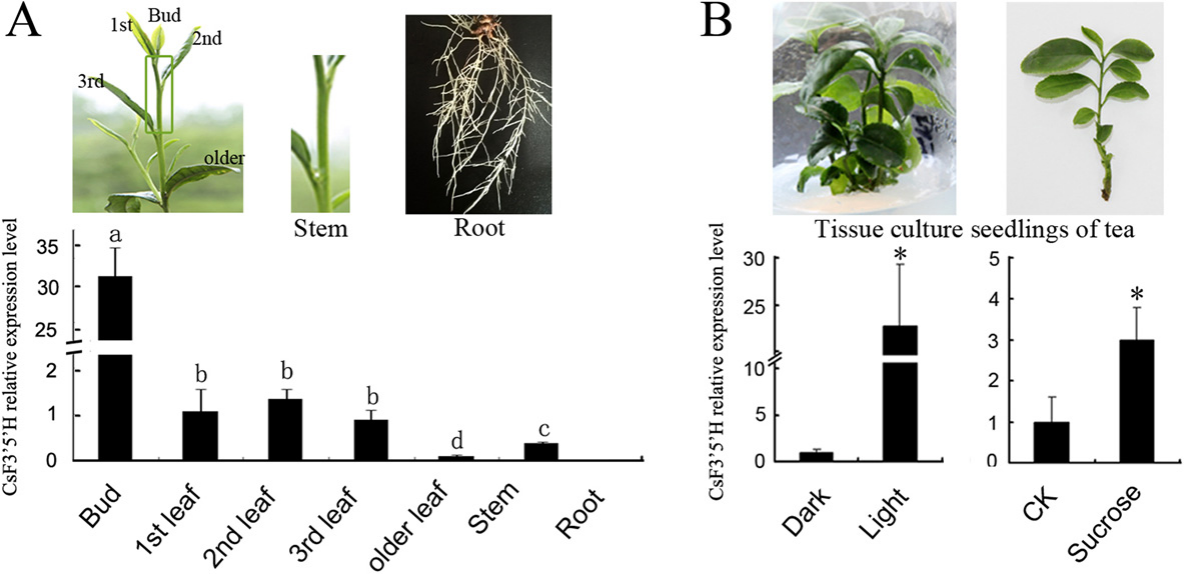
**Expression of CsF3′5′H in different tea tissues. (A)** Relative expression of *CsF3′5′H* in different tea tissues analyzed by qRT-PCR and Semi-quantitative RT PCR for Cs*F3′5′H* and GAPDH in different tea tissues. **(B)** Relative expression of *CsF3′5′H* in different light and sucrose conditions analyzed by qRT-PCR and Semi-quantitative RT PCR for GAPDH and Cs*F3′5′H* in different light and sucrose conditions. The data represent the mean ± SD from three independent measurements. The different letters (a, b, c, d) and *indicated the significant level at *P < 0.05*.

**Figure 4:**
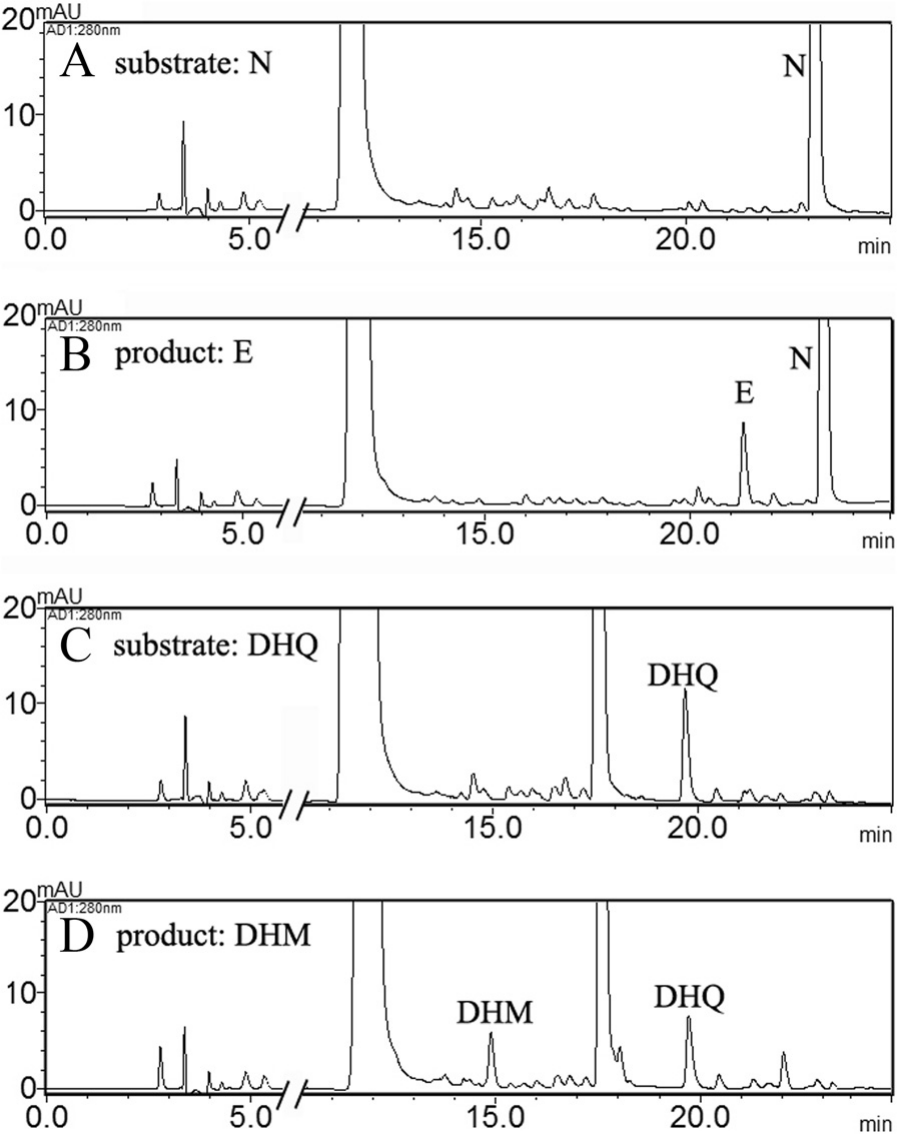
**HPLC chromatograms of flavanones or dihydroflavonols formation in CsF3′5′H assays with tea leaf enzyme. (A)** Reaction assay with substrate N of heat-denatured protein in the control treatment (the crude enzyme extract were heated to 100°C to inactivate enzyme activities); **(B)** Reaction assay with substrate N of the crude enzyme extract from the leave of tea; **(C)** Reaction assay with substrate DHQ of heat-denatured protein in the control treatment; **(D)** Reaction assay with substrate DHQ of the crude enzyme extract from the leave of tea.

The expression pattern of *CsF3′5′H* in tea was detected by qRT-PCR. The *GADPH* gene (accession number: FS952640), expected to show a constitutive expression pattern, was used as control [21]. *CsF3′5′H* expression was tissue specific, expressed highly in leaves and stem (Figure 3A), with transcripts peaking in the buds. We also assessed substrate specificity of crude extracts from tea leaves, measuring hydroxylation of N and Dihydroquercetin (DHQ), which yielded Eriodictyol (E) and Dihydromyricetin (DHM), respectively (Figure 4). The enzyme activities of these crude extracts were 0.072 and 0.023 pcat · g^−1^ protein, respectively. Surprisingly hydroxylation of N did not yield, 3′4′5′-hydroxylated product (5, 7, 3′, 4′, 5′-pentahydroxyflavanone, P).

Interestingly, *CsF3′5′H* transcripts were barely detected in the root, and the monomer and polymer of 3′ 4′ -dihydroxylated catechins (EC and ECG), but no 3′ 4′ 5′-trihydroxylated catechins, accumulated in Camellia sinensis roots [21], indicating that extremely low *CsF3′5′H* expression might directly lead to absence of B-ring tri-hydroxyl catechins in the root.

We used tissue culture seedlings, developed from the embryo of tea-seeds, to assess the direct influence of light and sucrose on *CsF3′5′H* expression. *CsF3′5′H* expression levels in light-exposed and sucrose-induced seedlings were significantly increased by 22.69 and 3.00-fold, respectively (Figure 3B), indicating that *CsF3′5′H* expression can be efficiently induced by light and sucrose.

### Functional analysis of the CsF3′5′H gene in *Nicotiana tabacum*

**Figure 5:**
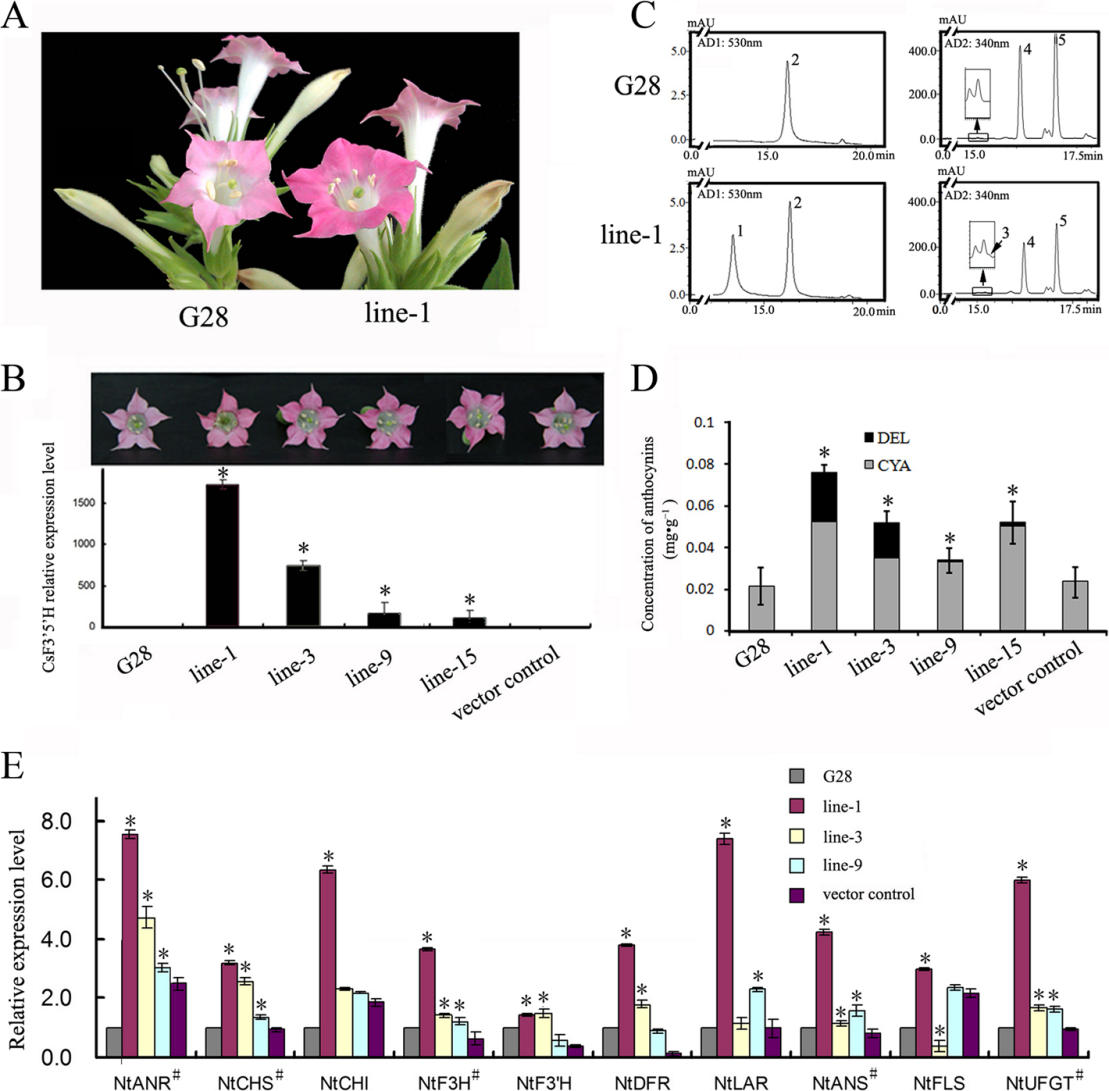
**Flower color after overexpression of CsF3′5′H and qRT-PCR of transgenic tobacco plants. (A)** Tobacco flowers of wild-type (CK) and CsF3′5′H transgenes (Line 1). **(B)** Tobacco flowers of wild-type (CK), CsF3′5′H transgenes (Line 1, 3, 9 and 15)and qRT-PCR for CsF3′5′H in flowers from CK and transgenic lines. **(C)** HPLC chromatograms of anthocyanidins (at 530 nm) and flavonol glacosides (at 340 nm) in tobacco flowers from CK and Line 1 (1: DEL; 2: CYA; 3: quercetin-3-O-rutinoside, 4: kaempferol-3-O-rutinoside). **(D)** Concentration of anthocyanidins in tobacco flowers from CK, CsF3′5′H transgenes (Line 1, 3 9, and 15) and vector control. The data represent the mean ± SD from three independent measurements. **(E)** qRT-PCR for flavonoid-related genes in tobacco flowers from CK, CsF3′5′H transgenes (Line 1, Line3 Line 9) and vector control. *indicated the significant level at P < 0.05. ^#^indicated the significant level compared between every detected lines versus CK (wild type and vector control).

The vector for constitutive expression of the 35S:*CsF3′5′H* gene was introduced into Tobacco ‘G28’ (*Nicotiana tabacum* ‘G28’), which lacks F3′5′H genes and has pink flowers [26]. About 20 independent transgenic tobacco plants were obtained. Most flowers from the transgenic plants exhibited a clear color change from pale pink of the host to magenta (Figure 5A).

The expression of *CsF3′5′H* in several transgenic lines with magenta flowers was detected by qRT-PCR, with *β-actin* (accession number: EU938079) used as reference gene (Figure 5B, E), and we found varying levels of *CsF3′5′H* gene expression in Glyphosate-resistant transgenic tobaccos. To investigate whether the flavonoid biosynthesis pathway was affected by over-expression of *CsF3′5′H*, the flavonoid pathway genes (*CHS* (chalcone synthase, accession number: AF311783)*, CHI* (chalcone isomerase, accession number: KJ730247)*, F3H* (flavanone 3-hydroxylase, accession number: AF036093)*, F3′H* (flavonoid 3′-hydroxylase, accession number: KF856279)*, DFR* (dihydroflavonol 4-reductase, accession number: EF421430)*, FLS* (flavonol synthase, accession number: DQ435530)*, ANS* (anthocyanidin synthase, accession number: JQ866631)*, ANR* (anthocyanidin reductase, accession number: XM_009786976)*, UFGT* (UDP-glycose flavonoid glycosyltransferase, accession number: GQ395697)) from *Nicotiana tabacum* were examined by qRT-PCR in wild type (G28) and transgenic lines. The expression levels of *CHS, F3H, ANS, ANR, UFGT* genes in transgenic lines significantly increased in comparison to the wild type and vector control (Figure 5E), suggesting that expression of these genes was stimulated by the over-expression of CsF3′5′H in transgenic lines.

The level of glycosylated flavonoids in flowers was assessed by reverse phase HPLC and LC-MS. 3′,5′-Hydroxylated flavonol glacoside (myricetin-3-O-rutinoside, MYR) was detected in the petals of the transgenic lines, but not in wild-type tobaccos (G28). However, the concentration of MYR in the flowers was too low to quantify (Figure 5C).

Petal pigments were extracted and chemically converted to anthocyanidins, for anglicizing the anthocyanin components by reverse phase HPLC. Petals expressing the CsF3′5′H gene contained a novel 3′,5′-hydroxylated anthocyanidin (delphinin, DEL) and increased cyaniding (CYA) derivative content. The ratio of delphinin to total anthocyanin compounds in transgenic tobacco plants reached a maximum of 31.09% (line-1, Figure 5D), and the average anthocyanin concentration in the petals of transgenic tobaccos was 1.51-fold higher than in wild-type plants, suggesting that CsF3′5′H encodes a protein with B-ring 3′, 5′-hydroxylation function, and that anthocyanin synthesis can be stimulated by CsF3′5′H over-expression in transgenic lines.

### Heterologous *CsF3′5′H* expression in yeast

**Figure 6:**
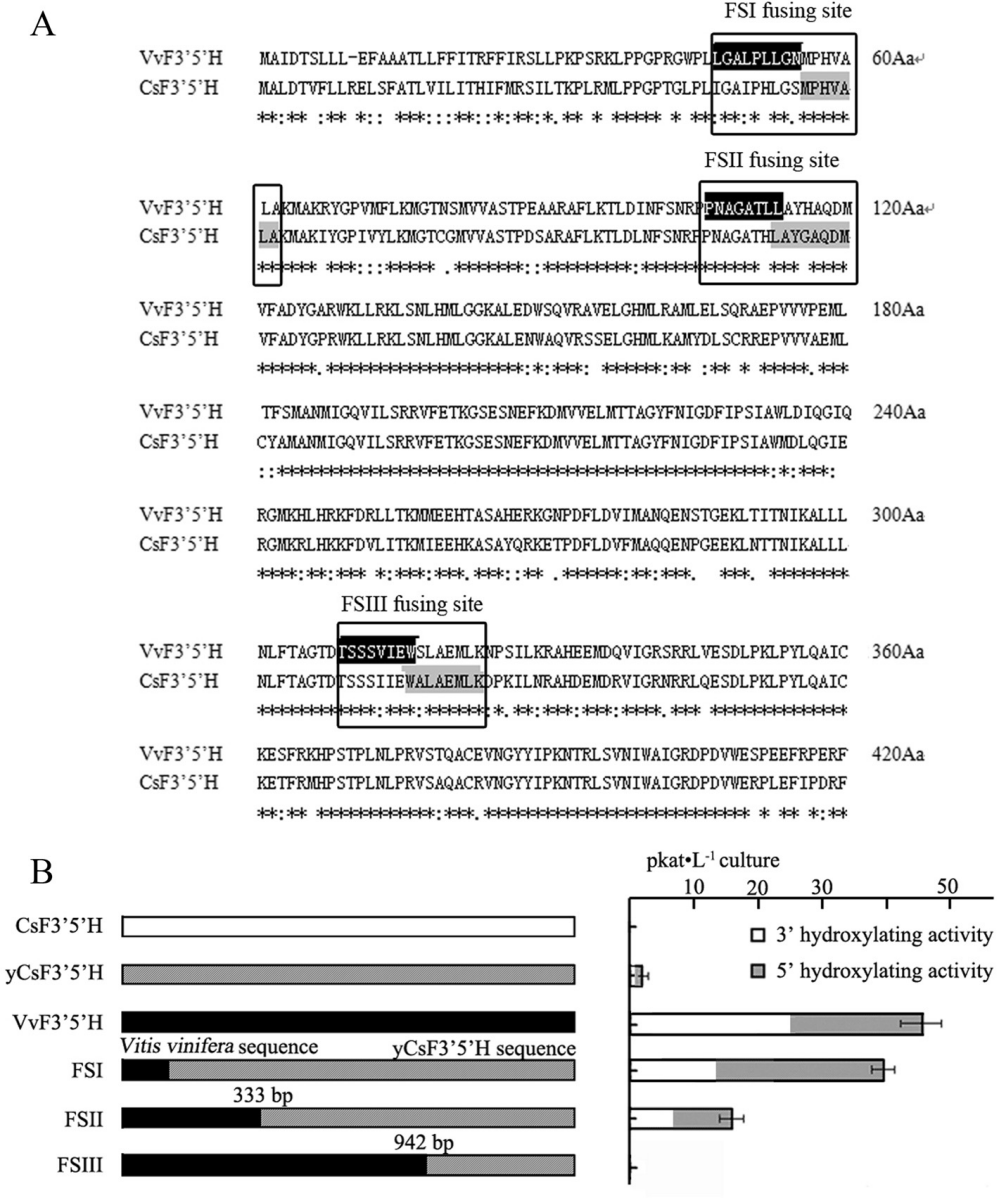
**Optimization of** ***CsF3′5′H*** **expression in** ***Saccharomyces cerevisiae*** **‘WAT11’. (A)** Comparison of amino acid sequences encoded by CsF3′5′H and VvF3′5′H proteins. The boxed regions represent fusion between CsF3′5′H and VvF3′5′H. **(B)** Primary structure schemes of expressed *CsF3′5′H* sequence variants and resulting expression strength expressed as *CsF3′5′H* activity · L^−1^ culture. Enzyme activity was expressed as pKat · L^−1^ culture. The data represent the mean ± SD from three independent measurements.

The yeast strain *Saccharomyces cerevisiae* WAT11, engineered to over-express the *Arabidopsis thaliana* P450 reductase [27], is a suitable heterologous host for P450 expression [11,28]. A pYES-DEST 52a CsF3′5′H vector was transformed into WAT11. However, these transgenic cells did not produce functional F3′5H protein (Figure 6), so the codon optimized yeast CsF3′5′H sequence (yCsF3′5′H) was designed and transformed into WAT11, resulting in only minimal activity of approximately 0.9 pkat · L^−1^ culture, with N as substrate.

Transgenic cells, harboring the *Vitis vinifera F3′5′H* (*VvF3′5′H,* NCBI cDNA accession number: XM_003632164) gene, achieved a high overall F3′5′H activity of 48.00 pkat · L^−1^ culture with N as substrate. With the predicted signal peptide, both *F3′5′Hs* were translated into precursor proteins and delivered to the ER. We hypothesized that imperfect recognition of the *Camellia sinensis* signal peptide might account for low expression levels detected in *Saccharomyces cerevisiae* cells, and tested this hypothesis by fusing *CsF3′5′H* with *VvF3′5′H* at three different points of the sequence based on amino acid sequence homology (Figure 6A). The 5′-sequences of yCsF3′5′H were replaced by VvF3′5′H at 55 Aa (Fusion sequence I, FSI), 153 Aa (Fusion sequence II, FSII), 308 Aa (Fusion sequence III, FSIII) respectively.

*Vitis vinifera* sequences were fused to *yCsF3′5′H* and cloned into the plasmid pYES-DEST 52a for transformation of WAT11 cells. The cells containing FSI, replaced at signal and leader peptide region, led to high F3′5′H activity in the range of 39.26 pkat · L^−1^ culture, a significant increase in comparison with the reference construct (yCsF3′5′H) (Figure 6B). These results indicated that *CsF3′5′H* signal peptide might be imperfectly recognized in *Saccharomyces cerevisiae* cells. The cells transformed with FSII also resulted in F3′5′H activity, albeit significantly less (in the range of 12.37 pkat · L^−1^ culture). Generally, overall activities of chimeras are often low, e.g. most chimeras between limonene 3-hydroxylase and limonene 6-hydroxylase achieve no, or less than 5% of that of wild type [29]. Unexpectedly, F3′5′H activity was undetected in cells transformed with FSIII. In comparison to *VvF3′5′H*, the FSIII fusion gene was only altered at the 3′-terminal sequence.

**Figure 7:**
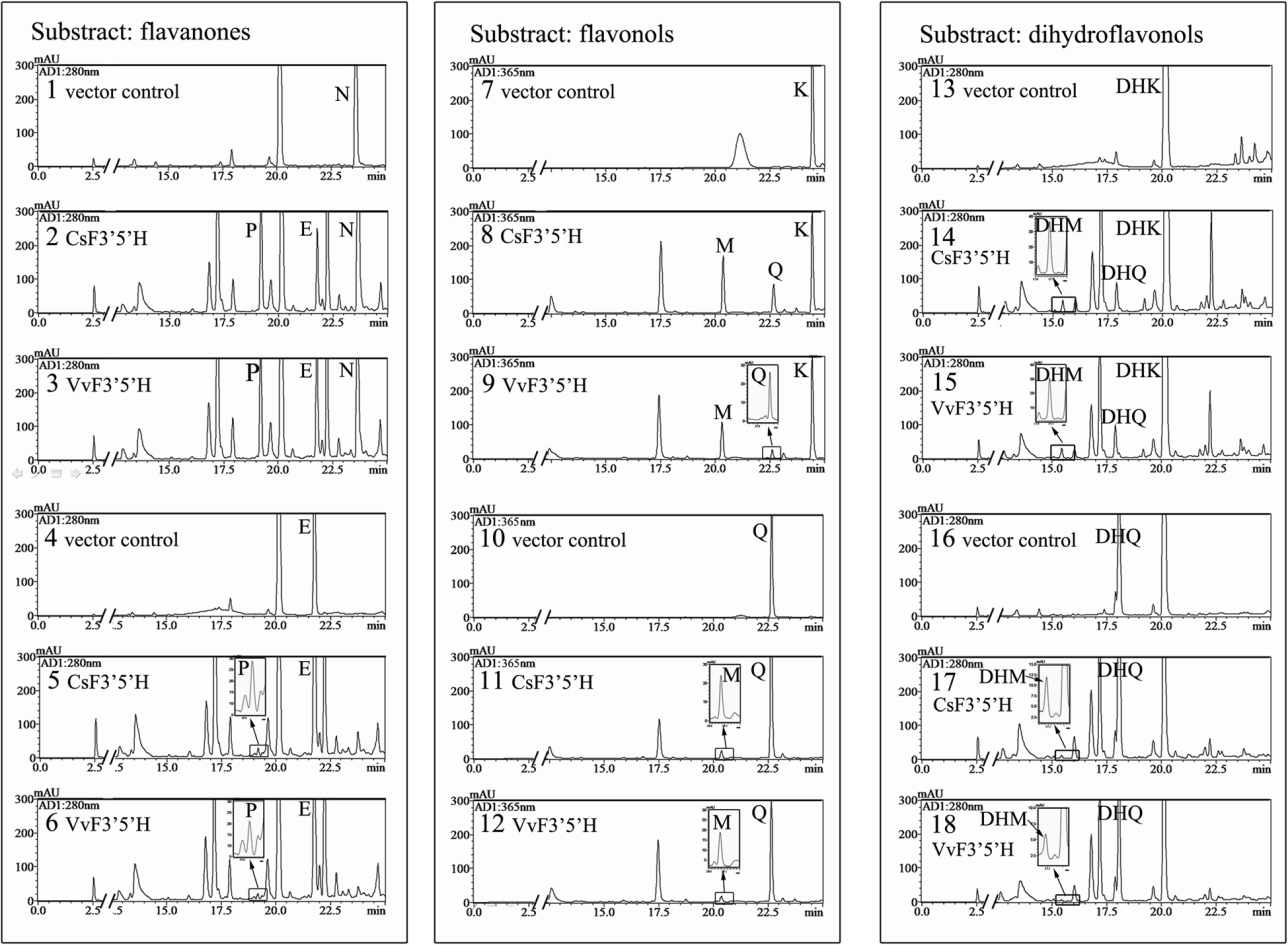
**HPLC chromatograms of products from pYES-dest52-** ***FS*** **and pYES-dest52-** ***VvF3′5′H*** **with flavanones, flavonols and dihydroflavonols as substrates.** HPLC chromatograms of products from pYES-dest52- FSI with N (2), E (5), K (8), Q (11), DHK (14) and DHQ (17) as substrates; HPLC chromatograms of products from pYES-dest52-VvF3′5′H with N (3), E (6), K (9), Q (12), DHK (15) and DHQ (18) as substrates; HPLC chromatograms of products from control treatment with N (1), E (4), K (7), Q (10), DHK (13) and DHQ (16) as substrates.

Finally, we assessed the substrate specificity of cells expressing FSI and *VvF3′5′H*. Based on previous findings and other intermediate compounds in the catechin synthesis pathway, we assessed catalysis of N, E, K, Quercetin (Q), DHK, DHQ, pelargonidin (PEL), CYA and C (Figure 7, Additional file 1: Figure S1, Table 1). WAT11 cells transformed with pYES-DEST 52a vector were used as controls. As observed with VvF3′5′H, FSI preferred B-ring 4′-hydroxylated compounds (including N, K and DHK) to 3′, 4′-hydroxylated compounds (including E, Q and DHQ). No activity was detected with PEL, CYA and C as substrates, in both transgenic cells. Both proteins displayed highest activities with N and significant activities with K and DHK, yielding 3′4′- and 3′4′5′-forms as products. Interestingly, for FSI with N as substrate, the ratio of 3′4′5′- to 3′4′-hydroxylated products (2.07:1) was significantly higher than for VvF3′5′H (0.98:1).

**Table 1:** **Accepted substrates and enzyme activity units for F3′5′H**

**Substrate**	**Modified-CsF3′5′H (FSI)**		**VvF3′5′H**		**Class**
	**3′-Hydroxyla-tion product (pKat · L** ^**−1**^ **)**	**5′-Hydroxylat-ion product (pKat · L** ^**−1**^ **)**	**3′-Hydroxylat-ion product (pKat · L** ^**−1**^ **)**	**5′-Hydroxylat-ion product (pKat · L** ^**−1**^ **)**	
Naringenin	12.79 ± 0.11	26.47 ± 1.08	24.27 ± 0.70	23.73 ± 0.85	Flavanone
Eriodictyol		0.13 ± 0.79		0.84 ± 0.13	
Kaempferol	4.58 ± 0.39	8.54 ± 0.40	2.84 ± 0.76	5.53 ± 0.44	Flavonol
Quercetin		0.58 ± 0.27		0.36 ± 0.19	
Dihydro-kaempferol	—	3.42 ± 0.54	—	2.69 ± 0.48	Dihydro-flavonol
Dihydro-quercetin		0.08 ± 0.03		0.05 ± 0.08	
Pelargonidin	—	—	—	—	Antho-cyanin
Cyanidin		—		—	
Catechin		—		—	Flavan-3-ols
Epicatechin		—		—	

Enzyme activity was expressed as pKat · L^−1^ culture. The data represent mean ± SD from three independent measurements. — indicates results below the detection limit.

**Figure 8:**
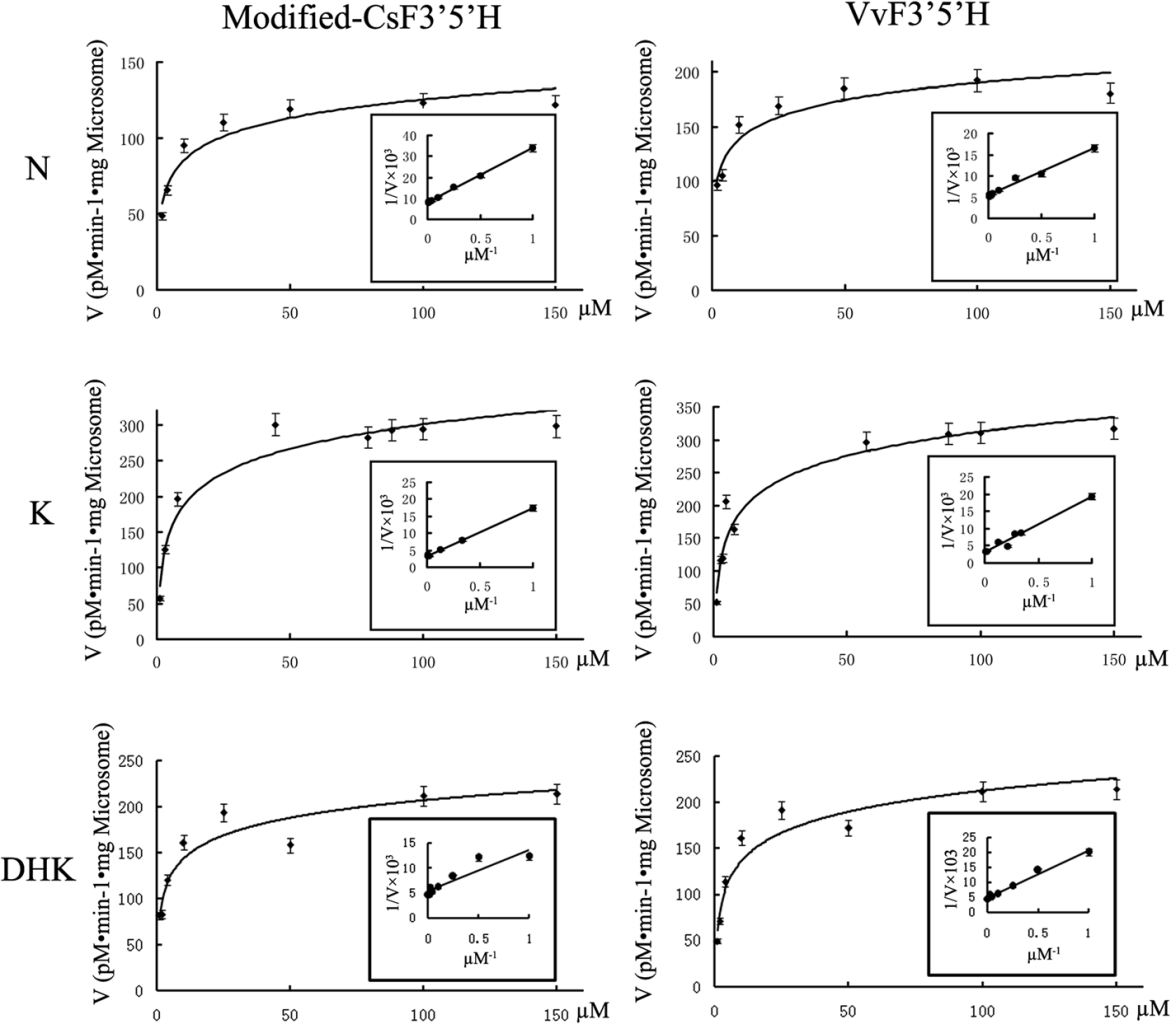
**Concentration dependence of F3′5′H observed in yeast microsomes.** F3′5′H-containing microsomes originating from transformed pYES-dest52-FSI and -VvF3′5′H cells were incubated in 50 mM phosphate buffer pH 7.0 at 28°C. The solid line represents the result of a multi-iterative fitting of experimental data using the Michaelis-Menten equation. Insert: Michaelis-Menten double-reciprocal plot. The data represent the mean ± SD from three independent measurements.

Microsomes from WAT11 cells transformed by pYES-DEST 52a -*FSI* and -*VvF3′5′H* were assayed for NADPH-dependent flavonoid 3′, 5′-hydroxylation with N, K and DHK as substrates. No activity was detected with microsomes from the control, pYES-DEST 52a-transformed cells. In contrast the K_m_ values of the microsome extracted from *FSI*-transformed cells, with N, K, and DHK as substrates, were 3.22, 4.33, and 3.26 μM, respectively (Table 2, Figure 8), indicating that N might be the optimum substrate for the CsF3′5′H enzyme. FSI achieved significantly higher K_m_ values than VvF3′5′H with K and DHK as substrates, but lower K_m_ values with N. However, the max reaction rates (V_max_) for FSI and VvF3′5′H with N as substrate were significantly lower than the values with K and DHK as substrates.

**Table 2:** **Comparison of steady-state kinetic parameters for cinnamate 4-hydroxylation in yeast microsomes**

**Substrate**	**Modified-CsF3′5′H (FSI)**		**VvF3′5′H**		**Class**
	**Km (μM)**	**Vmax (pM · min** ^**−1**^ **·mg** ^**−1**^ **Microsome)**	**Km (μM)**	**Vmax (pM ·min** ^**−1**^ **·mg** ^**−1**^ **Microsome)**	
Naringenin	3.22 ± 0.31	124.49 ± 10.11	2.03 ± 0.34	183.00 ± 11.02	Flavanone
Kaempferol	4.33 ± 0.19	306.00 ± 7.89	5.30 ± 0.71	327.00 ± 12.02	Flavonol
Dihydro-kaempferol	3.26 ± 0.25	219.00 ± 9.37	3.74 ± 0.54	220.18 ± 8.79	Dihydro-flavonol

Experiments were carried out in 50 mM phosphate buffer pH 7.0 at 28°C for 30 min. Protein concentration of *F3′5′H* -transformed yeast microsomes was 0.1 mg/ml in the reaction system. The data represent the mean ± SD from three independent measurements.

## Discussion

### The role of *CsF3′5′H* in catechin formation in tea leaves

All flavonoids are hydroxylated at the 4′ position of the B-ring. B-ring hydroxylation patterns determie the color of anthocyanins and thus have been extensively investigated in ornamental plants for color engineering. The F3′5′H gene is commonly known as the blue gene [30] and previous studies have shown that F3′5′H catalyzes the hydroxylation at the 3′ and 5′ positions of flavonoids to determine the hydroxylation pattern of the B-ring [28].

Flavonoids are important secondary metabolites in tea and account for 18 to 36% of the dry weight of fresh leaves and tender stem. 3′,4′,5′-trihydroxylated flavan-3-ols (catechins) are the most abundant flavonoids in tea leaves, present at significantly higher levels than 3′ 4′-dihydroxylated catechins. Catechins anabolic and catabolic processes are dynamic and subject to complex regulatory control, but the link between F3′5′H gene activity and relative catechin content is not well understood, due to the lack of easily assessable reporters.

Herein, we demonstrated that the *CsF3′5′H* gene is highly expressed in the leaves and stem, but expressed at extremely low levels in the root, as previously reported [25]. We have previously shown that the tea plant root lacks tri-hydroxyl groups in B-ring flavonols and flavan-3-ols, indicating that CsF3′5′H participates in the control of tri-hydroxyl flavan-3-ols synthesis in tea plant.

We found that *CsF3′5′H* gene transcripts peaked in the bud, and we were unable to explain the role of CsF3′5′H in the accumulation of leaf end-products. The content of most flavonoids such as galloylated catechins, PAs, and anthocyanidin were highest in the bud or first leaf and declined gradually with the leaf development [21]. These findings indicated that F3′5′H expression was closely associated with the accumulation of end-products of flavonoids in tea leaves.

CsF3′5′H expression was significantly increased after seven days of treatment with light or sucrose, indicated that *CsF3′5′H* expression can be efficiently induced by light and sucrose. Cloning analysis revealed that the *CsF3′5′H* gene promoter contains several light-responsive promoter elements (not shown), further indicating that light might be a key factor in the control of *CsF3′5′H* transcription*.*

### Anthocyanidin accumulation in CsF3′5′H transgenic tobacco

The main anthocyanin in the wild-type tobacco corolla is cyanidin [31]. As shown above, most transgenic plant flower petals contained delphinins. Interestingly, the cyanidin and delphinin content was significantly higher in transgenic tobacco plants than wild type plants, indicating that CsF3′5′H performs both 3′,5′- and 3′ -hydroxylation *in vivo*, in agreement with results of heterologous expression of F3′5′Hs in *Pericallis* × *hybrida* [10]*, Senecio cruentus* [32]*, Antirrhinum kelloggii* [9]*, and Solanum lycopersicum* [11]. However, the hydroxylation pattern of the B-ring cannot be elucidated by tobacco transgenic experiments. Flavonoid pathway, which is a complex metabolic network in plants, starts with general phenylpropanoid metabolism and leads to a myriad of end-products. The enzymes of flavonoid biosynthesis are likely to function as multienzyme complexes, which facilitate the direct transfer, or channeling of active sites [33]. Therefore, the overall concentrations of the intermediates, including free flavanones and flavanols, are extremely low in *vivo* [2].

*CsF3′5′H* transgenic tobacco plants produced deeper and redder flowers than wild-type plants. The qRT-PCR results indicated that the flavonoid pathway genes, including *CHS, F3H, ANS, ANR, UFGT*, could be stimulated by CsF3′5′H over-expression in transgenic lines. F3′5′H, a crucial microsomal cytochrome P450 enzyme in these pathways, may serve to anchor the complexes to the microsme membrane [33]. Therefore, our results indicate that over-expression of *CsF3′5′H* may stimulate metabolic flux toward anthocyanin products in tobacco petals by formatting more enzyme complexes.

The transgenic lines, however, did not produce blue flowers in this study. These findings demonstrated that blue flowers are not necessarily generated only by controlling the anthocyanin content [34]. Indeed, previous studies have reported that anthocyanidin content, co-pigments, metal ion type and concentration, pH of vacuoles, anthocyanin localization and shapes of surface cells all contribute to the final flower color [35]. However, our findings do reveal a clear impact of *CsF3′5′H* gene on flower phenotype. This gene might therefore be applied to molecular design of flower color in ornamental plants.

### Heterologous expression of CsF3′5′H in yeast

Heterologous expressions of CsF3′5′H in yeast were carried out to further confirm the catalytic position of CsF3′5′H enzyme in flavonoid pathways. To our knowledge, the *Camellia sinensis F3′5′H* gene has not been previously successfully expressed in yeast. For effective expression of CsF3′5′H in yeast, a codon optimized yeast CsF3′5′H sequence (yCsF3′5′H) was designed, but only minor activity was detected. Generally, the presence of an N-terminal signal peptide can translocate P450 proteins into the endoplasmic reticulum (ER). We further optimized *yCsF3′5′H* by replacing the N-terminal sequence with a signal peptide from the *VvF3′5′H* gene. Fortunately, transgenic cells expressing the fusion *F3′5′H* gene exhibited high F3′5′H activity, indicating that the signal peptide of *CsF3′5′H* might be imperfectly recognized in *S. cerevisiae* cells. Unexpectedly, another fusion gene (FSIII), only altered at the 3′-terminal sequence in comparison to *VvF3′5′H,* not achieving detectable F3′5′H activity. These results suggested that the region of F3′5′H conferring enzymatic activity might be located at the C-terminal of F3′5′H. Indeed, previous reports have suggested that the functional difference between *F3′H* and *F3′5′H* is determined by the C-terminal end [36].

F3′5′Hs have been shown to hydroxylate a broad range of flavonoid substrates, including N, DHK, K and apigenin, possibly allowing the formation of 3′,4′- and 3′,4′,5′-hydroxylated flavonoids. However, the optimum substrate for the F3′5′H enzymes needs to be further defined, *in vivo* and *in vitro*. F3′5′H enzymes from *Catharanthus roseus* and *Petunia x hybrida* have achieved highest activities with naringenin and apigenin [37], and N and DHK are equally hydroxylated by *Osteospermum hybrida* F3′5′H, whereas F3′H from *Gerbera hybrida* exhibits a clear substrate preference for N [36]. In contrast, the F3′5′H gene from tomato (*Solanum lycopersicum*) has a preference for naringenin, with a K_m_ value of 1.20 μM [11].

To assess substrate specificity of the modified CsF3′5′H (FSI), flavanones (N and E), flavonols (K and Q), dihydroflavonols (DHK and DHQ), anthocyanins (PEL and CYA) and catechin (C) were selected as substrates. 4′- hydroxylated flavanone (N) was the optimum substrate for the CsF3′5′H enzyme, and was effectively converted to both 3′4′- and 3′4′5′-forms. Interestingly, with N as substrate in FSI transgenic cells, the ratio of 3′4′5′- to 3′4′-hydroxylated products was significantly higher than in VvF3′5′H cells. Modified *CsF3′5′H* genes could thus tailor flavonoid metabolism, enhancing the yields of specific B-ring tri-hydroxyl products.

The broad substrate acceptance is consistent with the possibility that multiple paths lead to the same intermediates, and that competition could occur in *vivo*. The substrates used *in vivo* are mostly not yet precisely identified [37]. We also detected the B-ring hydroxyl reaction patterns of total enzyme extract from tea leaves. Interesting, with N as a substrate, the 3′4′5′-hydroxylated flavanone product (P) was undetected and only the 3′4′-hydroxylated product (E) was detected. It is not known whether the product P might be efficiently transformed into other end-products or the product E synthesized by another P450 gene (F3′H). Therefore, further analysis should be carried out to further describe the mechanism underlying B-ring hydroxylation *in vivo*.

## Conclusions

In this study, the *CsF3′5′H* gene was isolated from the tea cDNA library. Phylogenetic analyses revealed that the *Camellia sinensis F3′5′H* gene belongs to the CYP75A subfamily. qRT-PCR analysis indicated *CsF3′5′H* is highly expressed in the bud, but very little is expressed in the root. Over-expression of CsF3′5′H resulted in production of new delphinidin derivatives in the corollas of transgenic tobacco plants, increased the content of cyanidin derivatives and produced deeper and redder flowers in transgenic plants. Heterologous expressions of CsF3′5′H in yeast were carried out to demonstrate the function of CsF3′5′H enzyme *in vitro*. Heterologous expression of the modified y*CsF3′5′H* (FSI) in yeast revealed the 4′-hydroxylated flavanone naringenin to be the optimum substrate for the CsF3′5′H enzyme; naringenin was effectively converted into both 3′4′- and 3′4′5′-forms. Importantly, the ratio of 3′4′5′- to 3′4′-hydroxylated products was significantly higher in modified y*CsF3′5′H* transgenic cells than in VvF3′5′H cells. The findings reported here provide a basis for better understanding of the role of *Camellia sinensis* F3′5′H in B-ring hydroxylation of flavonoids, *in vitro* and *in vivo*.

## Methods

### Plant materials

Samples of *Camellia sinensis* cv. Shucazao (Variety Approval number: CHN20022008), were obtained from the experimental tea garden of Anhui Agricultural University in Hefei, China (north latitude 31.86, east longitude 117.27, altitude 20 m above mean sea level). Leaves were collected at five different stages (bud, 1st leaf, 2nd leaf, and 3rd leaf, older leaf), stem and root, snap frozen in liquid nitrogen and stored at −80°C.

Healthy tissue culture seedlings were used for light and sucrose induction experiments. Seedlings were cultured in normal light–dark cycle (light/dark: 14 h/10 h) in N6 medium containing 3% sucrose, and subcultured every 20 days by transferring about 5 g (fresh weight) to fresh medium. Six separate culture flasks were selected from the light and sucrose treatments. For light treatment, plates were exposed to 50 ± 5 μmolm^−2^ s^−1^ light (Cool white, 55 W, Philips, Netherlands) for 7 days, and culture flasks covered with aluminum foil were used as full darkness controls. For sucrose treatment, the seedlings were subcultured in the previously described medium or the previously described medium containing additional 90 mM/L sucrose for 7 days. Total RNA was isolated from leaves for quantitative real time polymerase chain reaction (qRT-PCR) in three independent experiments. The morphology of tea seedlings were captured with a Cannon 600D camera (Cannon, Japan).

The yeast strain (*Saccharomyces cerevisiae* cv. WAT11) and the tobacco variety (*Nicotiana tabacum* cv. G28), were kindly provided by Conagen Inc (Bedford, MA, USA) and University of Science and Technology of China (Hefei, Anhui, China), respectively.

### End-to-end PCR

The CsF3′5′H gene from the NCBI database was subjected to standard end-to-end PCR reactions, with the primers designed according to the cDNA sequence (synthesized by Invitrogen, Shanghai, China; Additional file 2: Table S1). The cDNA strands for end-to-end PCR were synthesized with Phusion® High-Fidelity DNA Polymerase (New England Biolabs, USA). PCR products were gel purified using the MiniBEST Agarose Gel Extraction Kit (Takara, DaLian, China), ligated into a pMD18-T vector, and transformed into *E. coli* DH5α competent cells for sequencing. The results were assembled using DNAMAN 7 software (Lynnon, Canada). Briefly, end-to-end PCR was performed under the following conditions: 98°C for 30 s, 30 cycles at 98°C for 30 s, 58°C for 10 s, 72°C for 40 s, and a final extension at 72°C for 10 min.

### Validation of expression by qRT-PCR

Total RNA was isolated from *Camellia sinensis* organs with RNAiso Plus (Takara, DaLian, China) and RNAiso-mate for Plant Tissue (Takara, DaLian, China), according to the manufacturers’ instructions.

All primers were blasted against the NCBI database to guarantee specificity. Values were normalized against the expression levels of the housekeeping gene glyceraldehyde-3-phosphate dehydrogenase *(GAPDH)* in tea plant [21] and actin in tobacco [38]. The first strand cDNA samples for qRT-PCR were synthesized with the PrimeScript® RT reagent Kit (Takara, DaLian, China). The PCR mixture contained cDNA template (approximately 0.01 μg/μL), 10 μL SYBR Green PCR Master Mix (Takara), and 200 nmolL^−1^ of each gene-specific primer in a final volume of 20 μL. Real-time PCR was performed using a CFX96™ optical reaction module (Bio-Rad, USA) as follows: 95°C for 30 s, followed by 40 cycles at 95°C for 5 s and 60°C for 30 s (58°C for 30 s for root) in 96-well optical reaction plates. The amplification specificity was verified by melting curve analysis (55–95°C). Data were expressed as mean value of three replicates, normalized against the expression levels of GAPDH or actin. The relative expression was derived by the 2^-ΔΔCt^ method. △C_T_ = C_T,__target_ -C_T,__internal standard_, −△△C_T_ = −(△C_T_, _target_ -△C_T_, _control_), where C_T,__target_ and C_T,__internal standard_ are cycle threshold (CT) values for targets and housekeeping genes, respectively.

### Transformation of tobacco plants with CsF3′5′H transgenes

The Gateway® Cloning System was used to construct the vectors provided by Prof Xiang [39] of the University of Science and Technology of China. *CsF3′5′H* PCR products were obtained by end-to-end PCR and ligated into pMD18-T vectors. The *CsF3′5′H* - pMD18-T plasmids were amplified in *E. coli* strain DH5α and used as PCR templates. The PCR primer pairs for linking the attB adaptors are listed in Additional file 2: Table S1. PCR products were purified, transferred to pMD18-T and confirmed by sequencing. The correct plasmid was cloned into the entry vector pDONR207 by Gateway® BP Clonase® Enzyme mix according to the manufacturer’s instructions (Invitrogen, USA). The resulting entry pDONR207- clones were selected on gentamycin plates and validated by restriction enzyme digestion. Entry vectors were then transferred into the Gateway plant transformation destination vector pCB2004 using Gateway® LR Clonase™ (Invitrogen, USA). Recombinant colonies pCB2004-Cs*F3′5′H* and control pCB2004 vectors were selected on kanamycin plates and validated by restriction enzyme digestion, followed by transformation into EHA105 by electroporation at 2500 V for about 5.5 ms.

A single colony containing each target construct was confirmed by PCR and used for genetic transformation of tobacco. EHA105-pCB2004-*CsF3′5′H* and EHA105-empty pCB2004 were inoculated in liquid LB medium containing 50 mg/L kanamycin and 50 mg/L spectinomycin. Cells were allowed to grow in the dark at 28°C, for 20–22 h at 200 rpm to OD_600_ = 0.6, then pelleted by centrifugation (6000 rpm, 10 min) followed by two washing steps with liquid MS medium containing 100 μmol/L acetosyringone (Sigma, R40456). The leaf disc approach was used for tobacco transformation, with 25 mg/L phosphinothricin selection [40].

### Construction of the yeast strain *Saccharomyces cerevisiae* ‘WAT11’ vector for CsF3′5′H expression

PCR products of *VvF3′5′H,* FS, FSII, FSIII were obtained by end-to-end PCR, gel purified, and ligated into pENTR™/TEV/D-TOPO vectors using Top cloning (pENTR™/TEV/D-TOPO® Cloning Kits, Invitrogen, USA). Then, the entry vectors pENTR-*VvF3′5′H,* pENTR-*CzyF3′5′H-1,* pENTR-*CzyF3′5′H-2*, and pENTR*-CzyF3′5′H-3* were cloned into the destination vector pYES-dest52 using Gateway® LR Clonase™ enzyme (Invitrogen, USA). The resulting pYES-dest52-*VvF3′5′H,* pYES-dest52-*FSI,* pYES-dest52-*FSII,* and pYES-dest52*-FSIII* were transformed into *Saccharomyces cerevisiae* WAT11 with Frozon-EZ yeast Transformation II™ (Zymo Research, USA).

Yeast cells were propagated at 28°C for 12 h in 10 ml SD-U liquid medium containing 20 g/l glucose, by inoculation of a single colony from a SGlu plate. The thalli collected were transferred into 10 ml SD-U medium containing 20 g/l galactose, and grown at 28°C for 5 h.

For substrate specificity experiments, N, E, DHK, DHQ, K, and Q were separately added into the yeast culture to a final concentration of 5 μM, and incubated at 28°C for 10 h. Reactions were terminated by sonication for 15 min and addition of ethyl acetate. Products from each reaction were extracted three times with 10 ml ethyl acetate, evaporated and re-dissolved in 150 μl methanol for HPLC analysis at 280–370 nm.

### Microsome preparation

Protein synthesis was indiced in the yeast culture by the addition of galactose and the microsomal yeast fraction was prepared with MgCl_2_ as described by Olsen *et al*. [11]. Protein quantities were estimated according to the Bradford method. The microsome was dissolved in 1.0 to 1.5 ml TEG (30% glycerol in 50 mM Tris–HCl with 1 mM EDTA) on ice. All buffers/solutions and centrifuge were pre-cooled to 4°C.

### Enzyme extraction from *Camellia sinensis*

About 2 g of tea leaves were homogenized under liquid nitrogen, and total protein was extracted with 0.1 molL^−1^ phosphate-buffered saline (PBS, pH 7.4) containing an equivalent amount of PVPP, then centrifuged at 15000 g for 10 min at 4°C. The supernatants were used to assess F3′5′H activity. Protein concentrations of enzyme extract were determined by spectrometric analysis using Coomassie Brilliant Blue G-250.

### Enzyme assays

All enzyme assays were carried out in phosphate buffer. In the multi-enzyme incorporative reaction system, the F3′5′H assay solution was incubated at 28°C for 30 min (for microsomes) or 1 h (for crude enzyme extract) in 100 mM phosphate buffer (pH 7.0) containing 1 mM NADPH, 1–300 μM substrates. Enzyme reactions were terminated by adding ethyl acetate. Products from each reaction were extracted three times with an equal volume ethyl acetate, evaporated and re-dissolved in 500 μl methanol for HPLC analysis at 280–370 nm.

### Flavonoid pigment preparation and analyses

Anthocyanin aglycones were extracted with 1.6 ml methanol containing 20% water from about 500 mg frozen tobacco flowers. After centrifugation at 6,000 g at 4°C for 5 min, supernatants were extracted three times by equal volume of ethyl acetate, and the extracts were added to 1/3 volume of 4 M HCl aqueous solution for acid-hydrolysis by heat treatment at 90°C for 1 h. Hydrolysates were tested by HPLC at 530 nm.

### HPLC and MS analyses

Mass spectra were acquired using the electrospray ionization in the negative ionization modes at fragmentation voltages of 175 V over the range of m/z 100 to 2000 on the UPLC-QQQ-MS/MS (Waters 2478, Waters Instruments) with drying gas flow of 12 L min^−1^, a drying gas temperature of 350°C, a nebulizer pressure of 35 psi, and capillary voltages of 3500 V.

The HPLC consisted of a quaternary pump with a vacuum degasser, thermostatted column compartment, autosampler and diode array detector (DAD). A Phenomenex Synergi 4u Fusion-RP80 column (5 μm, 250*4.6 mm) was used at a flow rate of 1.0 mL min^−1^. The column oven temperature was set at 25°C. The mobile phase consisted of 1% acetic acid in water (A) and 100% acetonitrile (B). The gradient increased linearly from 0 to 10% B (v/v) at 5 min, to 15% B at 15 min, 40% B at 20 min, 60% B at 22 min, and maintained at 10% B to 25 min. The DAD was set at 280 and 340 nm for real-time monitoring of the peak intensities. Ultraviolet (UV) spectra were recorded continuously from 200 to 600 nm for plant component identification.

Among the standards used, N, E, P, DHK, DHQ, and DHM were quantified at 280 nm, whereas K, Q, and myricetin (M) were quantified at 365 nm. All products, except P, were identified and quantified by mass spectrums (MS) and peak area compared with standards. Since standard samples of 5, 7, 3′, 4′, 5′-pentahydroxyflavanone were unavailable, P was identified with LC-MS, and its relative concentration was quantified using E as the molar equivalent. All samples were run in triplicate for both quantitation and multivariate statistical analysis.

### Bioinformatics and statistical analyses

The phylogenetic tree was constructed using protein sequences from several plant F3′5′H, F3′H, and Cinnamic acid 4-hydroxylase (C4H) enzymes retrieved from the NCBI database by ClustalW of MEGA5 (accession numbers are given in the phylogenetic tree, Figure 2). The phylogenetic tree was constructed according to the neighbor-joining method. Branches corresponding to partitions reproduced in less than 50% bootstrap replicates were collapsed. The evolutionary distances were computed using the p-distance method. Evolutionary analyses were conducted in MEGA5 (web page: http://www.megasoftware.net/).

Data were presented as the mean ± SD of three independent measurements. The statistical significance of differences between groups was determined with Student’s *t*-test using SPSS software (SPSS, Chicago, IL, USA). P < 0.05 was considered statistically significant.

### Supporting data

The data set(s) supporting the results of this article is (are) included within the article (and its additional file(s)). The cDNA and protein sequences from several plant F3′5′H, F3′H and C4H enzymes retrieved from the NCBI web page (http://www.ncbi.nlm.nih.gov/).

## Additional files

Additional file 1: Figure S1. UPLC-QQQ-MS analysis of products from *pYES-dest52-FS* assayed with different substrates. **(A)** MS analysis of E; **(B)** MS analysis of P; **(C)** MS analysis of Q; **(D)** MS analysis of M; **(E)** MS analysis of DHQ; **(F)** MS analysis of DHM. Additional file 2: Table S1. Sequences of primers used for cloning, fusion, and expression analysis of F3′5′H.

## Abbreviations

ANR: Anthocyanidin reductase
ANS: Anthocyanidin synthase
C: Catechin
C4H: Cinnamic acid 4-hydroxylase
CHI: Chalcone isomerase
CHS: Chalcone synthase
DFR: Dihydroflavonol 4-reductase
DHK: Dihydrokaempferol
DHM: Dihydromyricetin
DHQ: Dihydroquercetin
E: Eriodictyol
F3H: Flavanone 3-hydroxylase
F3′H: Flavonoid 3′-hydroxylase
F3′5′H: Flavonoid 3′,5′-hydroxylase
FLS: Flavonol synthase
K: Kaempferol
LAR: Leucoanthocyanidin reductase
M: Myricetin
N: Naringenin
P: 5, 7, 3′, 4′, 5′-pentahydroxyflavanone
Q: Quercetin
UFGT: UDP-glycose flavonoid glycosyltransferase
