# Supplementary material for: Functional analysis of Flavonoid 3′,5′-hydroxylase from Tea plant (Camellia sinensis): critical role in the accumulation of catechins
Source: BMC Plant Biol. 2014 Dec 10;14:347. doi: 10.1186/s12870-014-0347-7 (PMC4275960; doi:10.1186/s12870-014-0347-7)
Supplement: Additional file 1: Figure S1. — UPLC-QQQ-MS analysis of products from pYES-dest52-FS assayed with different substrates. (A) MS analysis of E; (B) MS analysis of P; (C) MS analysis of Q; (D) MS analysis of M; (E) MS analysis of DHQ; (F) MS analysis of DHM. [file 12870_2014_347_MOESM1_ESM.pdf]

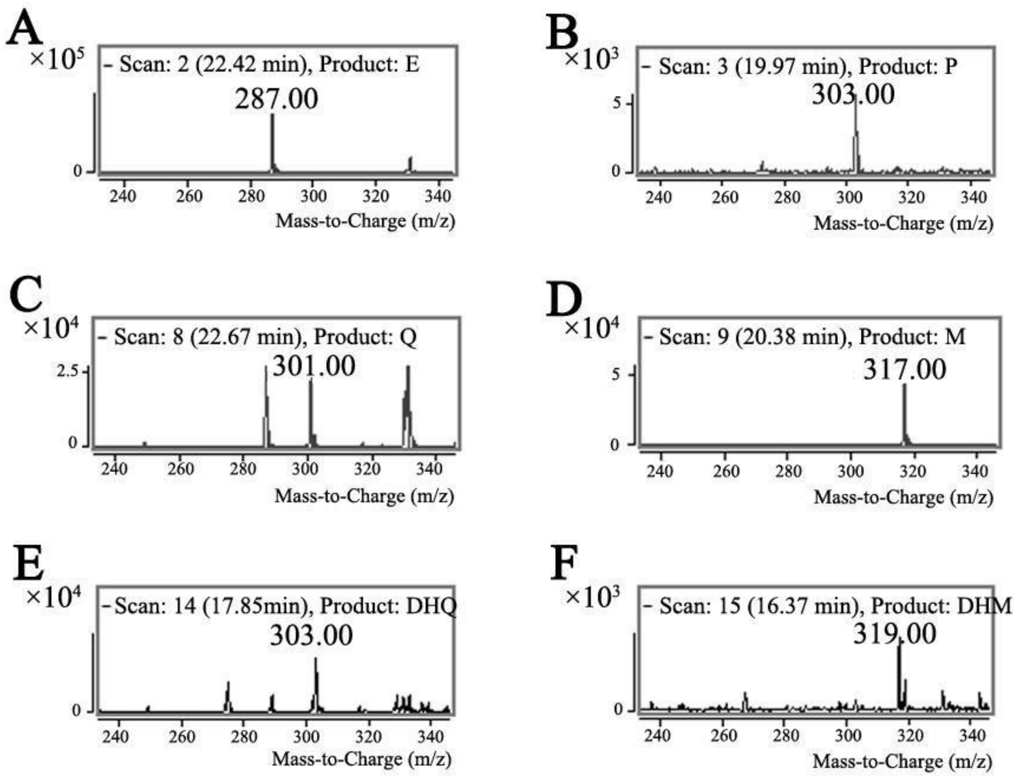

Additional file 1: Figure S1. UPLC-QQQ-MS analysis of products from *pYES-dest52-FS* assayed with different substrates. (A) MS analysis of E; (B) MS analysis of P; (C) MS analysis of Q; (D) MS analysis of M; (E) MS analysis of DHQ; (F) MS analysis of DHM.
