# Supplementary material for: Functional analysis of Flavonoid 3′,5′-hydroxylase from Tea plant (Camellia sinensis): critical role in the accumulation of catechins
Source: BMC Plant Biol. 2014 Dec 10;14:347. doi: 10.1186/s12870-014-0347-7 (PMC4275960; doi:10.1186/s12870-014-0347-7)
Supplement: Additional file 2: Table S1. — Sequences of primers used for cloning, fusion, and expression analysis of F3′5′H. [file 12870_2014_347_MOESM2_ESM.pdf]

Additional file 2: Table S1. Sequences of primers used for cloning, fusion, and expression analysis of *F3'5'H*

Primer name	Primer sequence（5'-3'）	Length of PCR product (bp)
CsF3'5'H- End-to-end PCR	S: AACCAGGTAGTGCTTTGCTTGCT<br>A:TTAAGCAGCATAAGCATTTGGAGG	1944
yF3'5'H- End-to-end PCR	F: ATGGCCCTAGACACCGTCTTCC<br>R: TTAAGCAGCATAAGCATTTGGAG	1533
FSI	F: ATGGCCATAGATACAAGCCTCTTGC<br>R: AGCTAGAGCAACATGTGGCATGTTACCTAGAAGAGGAAGAGCGCCG	183
FSII	F: ATGGCCATAGATACAAGCCTCTTGC<br>R: CATGTCCTGAGCACCGTAGGCTAAGAGTGTGGCACCGGCATTAGG	357
FSIII	F: ATGGCCATAGATACAAGCCTCTTGC<br>R: TCTTTCAACATTTCGGCTAGGGCCCACTCGATTACGCTCGATGAAGTG	968
GADPH- qRT-PCR	F:TTGGCATCGTTGAGGGTCT<br>R: CAGTGGGAACACGGAAAGC	206
CsF3'5'H- qRT-PCR	F: GGCAGACTCTGGGAAGAT<br>R: CGGCTACAAGAATCCGACCTA	133
VvF3'5'H-pENTR (BAE47007)	F:CACCATGGCCATAGATACAAGCCTCTTGC	
NtActin(AY179605)	F: TCGGAATGGAAGCTG<br>R: TGGTACCACCACTGAGGACA	111
CsF3'5'H-3' RACE	outer :ACGGCTACAAGAATCCGACCTA<br>inner: TAGAATGGGGATTGTGCTTGTTG	541
CsF3'5'H-5' RACE	outer : GCTTGGGCAGCGACTCTGGGAAGAT inner: CAGCAGCGAATAAGTTCAAGAGTAG	1428
CsF3'5'H-attB	F:GGGGACAAGTTTGTACAAAAAAGCAGGCTATGGCCCTAGACACGGTCTTCCTGCT<br>R:GGGGACCACTTTGTACAAGAAAGCTGGGTTTAAGCAGCATAAGCATTTGGAGGC	1591
